# What does lack of language lateralization signify? Evidence of fluctuating asymmetry rather than hemispheric equipoise on non-lateralized tasks

**DOI:** 10.1098/rsos.240495

**Published:** 2024-08-14

**Authors:** Dorothy V. M. Bishop

**Affiliations:** ^1^ Department of Experimental Psychology, University of Oxford, Oxford OX2 6GG, UK

**Keywords:** laterality, handedness, brain, language, bilateral, fluctuating asymmetry

## Abstract

In a study of patterns of language laterality in left- and right-handers, Woodhead *et al*. (Woodhead ZVJ, Thompson PA, Karlsson EM, Bishop DVM. 2021 *R. Soc. Open Sci*. **8**, 200696. (doi:10.1098/rsos.200696)) noted that several tasks showed no bias to the left hemisphere in left-handed individuals. This might appear to suggest that these functions were mediated by the two hemispheres working together equally—what can be termed ‘hemispheric equipoise’. Here, I consider an alternative possibility that individuals show lateral bias on these tasks, but the bias can occur to either the left or right—a form of fluctuating asymmetry. Further analysis of the distributions of data from individuals in Woodhead *et al.* is compared with simulated data. The pattern of results suggests that the impression of hemispheric equipoise may be an artefact of reliance on group data: even though the group mean does not differ from zero, a high proportion of individuals are biased to the left or right.

## 1. Introduction

This study is a further update of Woodhead *et al*. [1] published in *Royal Society Open Science*. That study compared patterns of language laterality on six different tasks in 31 left-handers and 43 right-handers, finding stronger left-lateralization in right-handers and evidence for two separate language factors in left-handers.

Here, I follow up on an incidental observation from that study concerning tasks with reduced or absent language lateralization. The test battery included one task (Syntactic Decision) where the mean laterality index (LI) showed no lateral bias in either left- or right-handers, plus a further three tasks (Phonological Decision, Semantic Decision and Sentence Comprehension) that were not lateralized in left-handers. Our previous reports of this dataset were focused on the characteristics of language tasks that were lateralized or non-lateralized [1]. Here, the focus turns to the nature of individual differences in asymmetries on non-lateralized tasks.

A key distinction will be drawn between ‘hemispheric equipoise’, where both hemispheres participate equally to perform a task, and fluctuating asymmetry, where the population contains a mixture of individuals who are biased to the left- or right-hemisphere at random. One may question whether the new term ‘hemispheric equipoise’ is needed, given that it is similar to the well-established concept of bilateral language processing. However, ‘bilateral language’ has historically been used rather loosely to correspond to lack of asymmetry in either individual or group data. Here, ‘hemispheric equipoise’ has the specific connotation that both hemispheres are engaged together in a function *within an individual,* without a significant bias to one side or the other.

Language tasks that are not lateralized are largely ignored in the literature on brain asymmetry, but I argue here that they do have the potential to throw light on the nature of cerebral lateralization. At first glance, a lack of lateral bias might seem to indicate that for these tasks there is ‘hemispheric equipoise’, with both hemispheres participating equally in task performance, and any variability is due solely to random noise (i.e. measurement error). However, if that were the case, we would not expect to see significant test–retest correlations for LIs on these tasks, nor should we observe significant correlations between the LIs with other tasks. As reported in figure 3 of Woodhead *et al*. [1], the test–retest correlations for left-handers were 0.80, 0.76, 0.85 and 0.85 for these four tasks, and there were also healthy cross-task correlations, of around 0.5 to 0.7.

This suggests that the group data may contain a mixture of individuals, some of whom are lateralized to the left and others to the right. This would give a radically different interpretation—rather than hemispheric equipoise, we would conclude that for this phenotype there is a form of fluctuating asymmetry, where the function is lateralized to left or right, with the direction of asymmetry in a given individual being determined by chance.

The current study uses simulations to predict distributions of LIs from different models of language lateralization and compares these with observed distributions, concluding that hemispheric equipoise is much less common in individuals than might be suggested by the group data. Rather, we see a high proportion of left- and right-biased individuals in the population.

### 1.1. Formal modelling of language lateralization

In the first paper in this series, Woodhead *et al*. [2] noted that we can simulate LIs as the sum of a set of terms representing:

*t* = mean lateral bias associated with the task,

*p* = mean lateral bias of the person,

*e* = random error, and

*x* = interaction between person and task effects

Here, I focus on the case of bilateral tasks, where the population mean does not differ from zero, so we can drop the terms involving task effects.

The aim is to simulate data similar to that used by Woodhead *et al*. [1], where people were tested on two occasions on a task, and on each occasion, 15 test trials were given. Then for person *i*, in session *j*, on trial *k*


$$LI_{ijk}=p_{i}+e_{ijk}.$$

When simulating data from this model, we need to specify how to simulate the distribution of *p*_*i*_ in the population. The simplest assumption is that *p*_*i*_ is normally distributed with a mean of zero. In that case, most people have a true underlying laterality close to zero, but the extent to which they depart from bilaterality constitutes a stable individual difference.

Predictions from this model will depend on the variance of *p* and *e*. If the variance of *p* is large relative to the variance of *e*, then stable individual differences in laterality will be apparent, and we will see, on the one hand, substantial variation between individuals and, on the other hand, stability of the LI from session 1 to session 2. If the variance of *p* is small relative to the variance of *e*, then the principal factor determining observed laterality will be noise, and the test–retest reliability of the LI will be low.

In the limiting case, *p*_*i*_ is zero, so there are no stable individual differences in laterality—both cerebral hemispheres participate equally, and the only source of variation is random noise, so


$$LI_{jik}=0\;+e_{ijk}.$$

A simple simulation of this model in the R programming language [3] is available on the Open Science Framework. The *rnorm* function, which takes arguments of *n*, *mean* and *sd*, generates random normal deviates twice in the simulation: first, we simulate values of *p* for a specific person with the code


p⋅i<−rnorm(n=1,mean=0,sd=p.sd),

that is, generate a single random number with a mean of 0 and a variance of *p.sd*. If *p.sd* is zero, there are no individual differences, and *p.i* is always zero.

The second occurrence of the rnorm function is to generate the LIs for each trial for a given person,


LI.ik<−rnorm(n=ntrial,mean=p⋅i,sd=e⋅sd)

The value of ntrial is set to 15, to simulate data comparable to the real data (see below). The previously computed *p.i* variable is used to specify the mean LI for an individual person, and *e.sd* specifies the standard deviation of the error.

In theory, when modelling test–retest data, it would be possible to include an additional term corresponding to test session, but we have never found any systematic effect of session on LIs from functional transcranial Doppler ultrasound (fTCD), so here we assume no session effect and simply model test–retest data by running the simulation twice to generate two sets of data.

**Figure 1.**
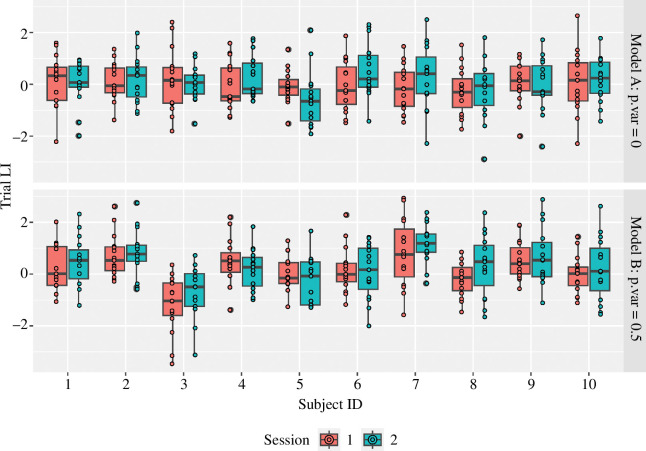
Simulated data for 10 participants. In model A, the LI of each trial is determined by chance. In model B, each participant has a specific bias (simulated as a random normal deviate with a mean of 0 and s.d. of 0.5).

Figure 1 shows the distribution of observed LIs for two test sessions each of 15 trials for 10 simulated subjects. In both cases, the value of *e.sd* was set to 1. For model A, the value of *p.sd* was 0, whereas for model B, it was 0.5. Each boxplot shows the LIs for the 15 individual trials obtained in one session. The value of *e.sd* determines the spread of LIs within a given subject and session (i.e. reflecting the range seen within each plot). The value of *p.sd* determines the variation in the means of the boxplots (reflecting systematic variation from person to person). In both models, the average mean LI across all individuals is zero, but model B shows systematic variation from person to person.

The formalization of the model captures a very simple point, namely that for model A, there is no meaningful individual variation in the LI: everyone shows hemispheric equipoise with a mean of zero. For model B, some people are reliably left-lateralized and some are reliably right-lateralized, to a greater or lesser extent. This will be reflected in measures of test–retest reliability. For model B, where there is a significant *p* term, the test–retest correlation for simulated data is 0.79, whereas for model A, it is −0.045.

**Figure 2.**
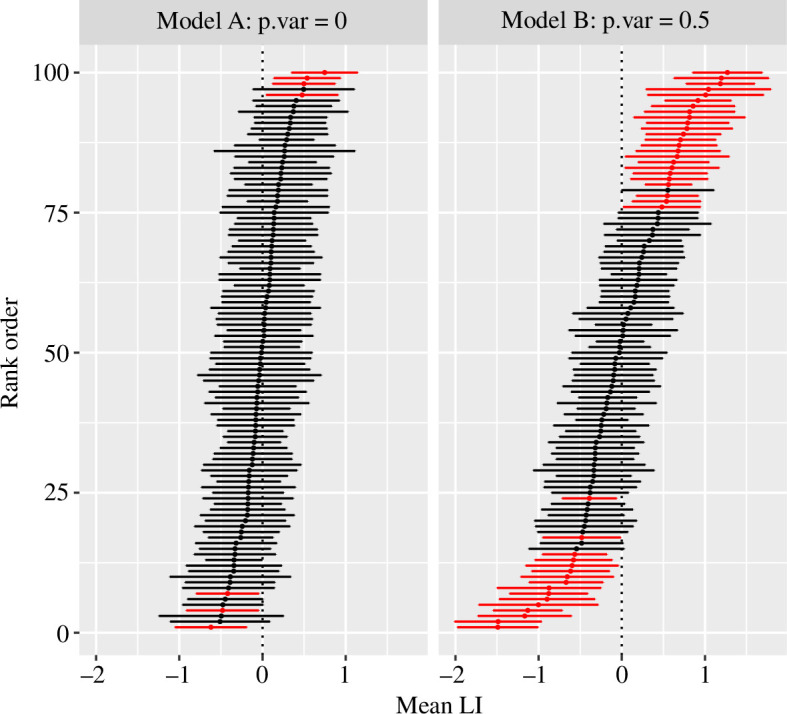
Ranked forest plots for models A and B. The LIs are averaged for each simulated participant and then displayed in rank order, with the most leftward biased at the top (positive LI) and the most rightward biased at the bottom. The central points show the mean LI, and the horizontal fins show the 95% confidence intervals (CIs). Lines are shown in red for those where the CI does not include zero: i.e. where the individual is significantly lateralized.

Even if we do not have retest data, we can distinguish the models in terms of the stability of estimates of LI across trials within a session. Each individual in each session can be classified as lateralized or unlateralized, depending on whether the 95% confidence interval around the LI estimate includes zero. Figure 2 shows the rank-ordered LIs from 100 simulated participants for each model, with red bars indicating those that are significantly lateralized.

Figure 2 shows ranked forest plots, i.e. a stacked display of rank-ordered mean LIs with their confidence intervals as fins. For model A, where only random error determines the individual mean LIs, around 95% of cases are categorized as being unlateralized on the basis that the 95% confidence interval around the mean LI encompasses zero. For model B, only 61% of cases are categorized as unlateralized. We can see that the high proportion of cases that is lateralized on this criterion is an indicator that there are stable individual differences in laterality.

### 1.2. Comparison of simulated distributions with empirical data on non-lateralized tasks

We can now compare ranked forest plots with the empirical data from Woodhead *et al*. [1]. For full details of methods, see the original paper; here a brief summary is given, taken from the original text.

## 2. Methods

There were 74 participants (43 right-handed, 31 left-handed) who were given a battery of six language tests on two occasions separated by 3 days to 6 weeks. Handedness was assessed by self-report. Simultaneous bilateral fTCD was used to measure the cerebral blood flow velocity (CBFV) in the left and right middle cerebral arteries as the participant performed the language tasks. The difference between left and right CBFV during a prespecified period of interest was averaged to give a LI. Note that this is a difference score, without any restriction of range, rather than a conventional LI (which is bounded at −1 and 1).

The measures were chosen to tap a broad range of language functions, encompassing language production and receptive language in tasks that placed demands on phonology, semantics and syntax. They were as follows:

— A. List Generation, which required production of automatic speech (counting, reciting the days of the week or months of the year) in response to a picture;
— B. Phonological Decision, where participants decided whether the names of two pictures rhymed; although this task did not involve explicit speech production, it was designed so that it would require covert speech for successful performance;
— C. Semantic Decision, where participants decided whether two pictures were semantically related;
— D. Sentence Generation, which required production of a meaningful sentence to describe a picture;
— E. Sentence Comprehension, where participants decided which of two pictures matched a spoken sentence;
— F. Syntactic Decision, where participants decided whether a sequence of words and non-words formed a plausible ‘jabberwocky’ sentence with correct syntactic structure.

All stimulus materials for the tasks are available on the Open Science Framework (https://osf.io/8s7vn/). There were 15 trials of each task per session, administered in separate runs. All tasks shared a common structure with an inter-stimulus interval of 33 s. Trials started with a 3 s ‘Clear Mind’ prompt, followed by the language task for 20 s, and ended with 10 s of rest.

## 3. Results

Given that the impetus for this analysis was the lack of language lateralization for left-handers in group data, we focus on the four tasks that were singled out for comment by Woodhead *et al.*, namely: (B) Phonological Decision, (C) Semantic Decision, (E) Sentence Comprehension and (F) Syntactic Decision. For tasks B, C and E, the group data indicated that left-handers were not lateralized, whereas right-handers were left-lateralized. For task F, both left and right-handers showed a non-lateralized profile at the group level.

**Figure 3.**
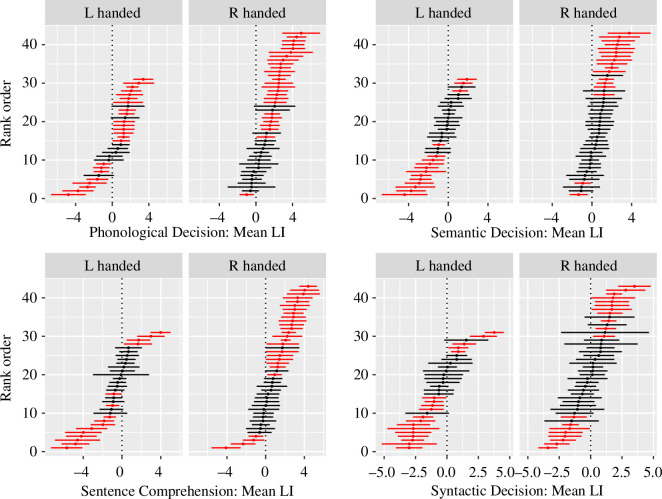
Ranked forest plots from session 1 for left- and right-handers on four tasks. The mean LIs for each participant displayed in rank order, with the most leftward biased at the top (positive LI) and most rightward biased at the bottom. The central points show the mean LI, and the horizontal fins show the 95% confidence intervals (CIs). Lines are shown in red for those where the CI does not include zero: i.e. where the individual is significantly lateralized.

Figure 3 shows ranked forest plots from session 1 for left- and right-handers on these four tasks. Session 2 data are very similar, and the plots for these are available on the Open Science Framework [4], together with plots for tasks A (List Generation) and D (Sentence Generation), which were significantly left-lateralized in both left- and right-handers.

By inspection we can see that for all four tasks, the proportion of individuals with significant lateralization of the LI (shown in red) is far higher than the 5% predicted by a model with no stable individual variation. Around 50% of left-handers show significant lateralization, with a mixture of left- and right-sided bias. Where right-handers have a lateral bias, it is predominantly to the left, except on the Syntactic Decision task (F), where a relatively high proportion of participants are lateralized, either to the left or to the right, in both left- and right-handed groups.

## 4. Discussion

The data from Woodhead *et al.* [2] provided an unexpected result that provides a novel perspective on the nature of cerebral asymmetry: there are some language tasks where the population bias to left-sided processing is absent or greatly decreased, but which nevertheless give reasonably stable LIs in individuals.

This analysis stresses the importance of distinguishing between group data and individual data when considering whether a language function is lateralized. Four of the six tasks in the language battery were not significantly lateralized in left-handers, as determined by comparing the group mean LI with zero. Although it might be tempting to conclude that these are functions that are mediated by both hemispheres contributing equally, the LIs from individuals show that this is not the case: a high proportion of participants showed a significant bias to one side for these tasks, but in left-handers (and in both left- and right-handers for Syntactic Decision) the bias was at least as likely to favour the right as the left hemisphere.

The distinction between the picture seen in group and individual data is reminiscent of a discussion by McManus [5], who used the example of Kartagener’s syndrome in which there is an association of situs inversus totalis (where the heart is on the right rather than the left) and chronic bronchiectasis. As he noted, ‘we would not describe the bronchiectatics as being less lateralised, in the sense that on average their hearts were nearer to the midline than in a control group, but rather we would describe the bronchiectatic population as having a higher proportion of an atypical subtype who are exact mirror-images of the normal individuals’ (p. 211). Of course, the location of the heart has to be to the left or right, whereas with the brain, it is not implausible that the two sides might work cooperatively to perform some functions with an equal division of labour. Furthermore, the notion that a proportion of people have ‘bilateral language’ has a long history on the basis of Wada testing, where one finds individuals with equivalent disruption of language by injection of both left and right hemispheres (see [6] for review).

The current analysis emphasizes the difficulty of distinguishing true lack of lateralization from error of measurement. Figure 3 shows many individuals who did not meet the criterion for significant lateralization, but they are not sharply divided from those who are lateralized, and the selection of a different cut-off would have included more or less of these as lateralized. In a different study, our group found when reanalysing language laterality data from functional magnetic resonance imaging (fMRI) that the proportion of individuals categorized as having bilateral language declines when we adopt analytic methods designed to reduce measurement error [7]. In a similar vein, it is noteworthy that when Janecek *et al*. [8] compared language laterality from fMRI and Wada testing in the same patients, discordant findings were most common in those categorized as having bilateral language. This suggests a provocative working hypothesis that true hemispheric equipoise with both hemispheres working together equally could be the exception rather than the rule in language tasks that have traditionally been regarded as ‘bilateral’. It will be of interest to test this with other datasets that have laterality data from fTCD and/or fMRI and to extend beyond language to other domains.

A final point to emerge from this reanalysis is that our understanding of language lateralization may, paradoxically, be informed by studying tasks in which there is no asymmetry at the population level. Most studies of lateralization have, naturally enough, focused on tasks showing clear left lateralization at the population level, and these have yielded a strong consensus about some basic associations, e.g. that around 95% of right-handers and 70% of left-handers are left-lateralized for language [9]. This pronounced asymmetry, however, creates difficulties for those interested in individual differences, because cases of atypical asymmetry are relatively rare. There is far less agreement about atypical variants in cerebral asymmetry: are these extreme points on a continuum, or are there distinct categories, and if so, how many subtypes are there? [10]. The tasks studied here provide one route to start addressing these issues by looking at individual variation in the absence of a strong population bias to asymmetry.

## Data Availability

The data used in this paper were previously deposited here: (doi:10.17605/OSF.IO/5BZ4J). The analysis script and platestack plots for all tasks and sessions are available [4].
